# Multiscale Medical Image Fusion in Wavelet Domain

**DOI:** 10.1155/2013/521034

**Published:** 2013-12-22

**Authors:** Rajiv Singh, Ashish Khare

**Affiliations:** Department of Electronics and Communication, University of Allahabad, Allahabad 211002, India

## Abstract

Wavelet transforms have emerged as a powerful tool in image fusion. However, the study and analysis of medical image fusion is still a challenging area of research. Therefore, in this paper, we propose a multiscale fusion of multimodal medical images in wavelet domain. Fusion of medical images has been performed at multiple scales varying from minimum to maximum level using maximum selection rule which provides more flexibility and choice to select the relevant fused images. The experimental analysis of the proposed method has been performed with several sets of medical images. Fusion results have been evaluated subjectively and objectively with existing state-of-the-art fusion methods which include several pyramid- and wavelet-transform-based fusion methods and principal component analysis (PCA) fusion method. The comparative analysis of the fusion results has been performed with edge strength (*Q*), mutual information (MI), entropy (*E*), standard deviation (SD), blind structural similarity index metric (BSSIM), spatial frequency (SF), and average gradient (AG) metrics. The combined subjective and objective evaluations of the proposed fusion method at multiple scales showed the effectiveness and goodness of the proposed approach.

## 1. Introduction

The development of multimodality medical imaging sensors for extracting clinical information has influenced to explore the possibility of data reduction and having better visual representation. X-ray, ultrasound, magnetic resonance imaging (MRI), and computed tomography (CT) are a few examples of biomedical sensors. These sensors are used for extracting clinical information, which is generally complementary in nature. For example, X-ray is widely used in detecting fractures and abnormalities in bone position, CT is used in tumor and anatomical detection, and MRI is used to obtain information about tissues. Thus, none of these modalities is able to carry all complementary and relevant information in a single image. Medical image fusion [1, 2] is the only possible way to combine and merge all relevant and complementary information from multiple source images into single composite image which facilitates more precise diagnosis and better treatment.

The basic requirements for image fusion [3] are as follows: first, fused image should possess all possible relevant information contained in the source images; second, fusion process should not introduce any artifact or unexpected feature in the fused image.

Image fusion can be classified into three categories: pixel level fusion, feature level fusion, and decision or symbol level fusion [4]. Pixel level fusion [5] deals with information associated with each pixel and fused image can be obtained from the corresponding pixel values of source images. In feature level fusion [6], source images are segmented into regions and features like pixel intensities, edges, and textures, are used for fusion. Decision or symbol level fusion [7] is a high-level fusion which is based on statistics, voting, fuzzy logic, prediction and heuristics, and so forth. For present work, we have considered pixel level image fusion due to its simple computation and understanding.

Spatial and transform domains [8] are the two fundamental approaches for image fusion. In spatial domain fusion, the fusion rule is directly applied to the intensity values of the source images. Averaging, weighted averaging, principal component analysis (PCA) [9], linear fusion [10], and sharp fusion [11] are a few examples of spatial domain fusion scheme. One of the major disadvantages of spatial domain fusion method is that it introduces spatial distortions in the resultant fused image and does not provide any spectral information. These spatial distortions have been observed in sharp fusion [11] method for medical images and reported in [12]. Since medical images are generally of poor contrast, the spatial information should be preserved in the medical images without introducing any distortion or noise. These requirements of medical images are better preserved in transform domain fusion.

Therefore, transform domain fusion techniques have been used for fusion to overcome the limitations of spatial domain fusion methods. Pyramid and wavelet transforms [13] have been used for multiscale fusion under category of transform domain methods. Transform domain fusion is performed by decomposing source images into transformed representations followed by application of fusion rules to the transformed representation. Finally, fused image is obtained by inverse transformation. Pyramid and wavelet transforms are the mostly used transforms for image fusion. Several pyramid transforms like Laplacian pyramid [14], gradient pyramid [15], contrast pyramid [16], ratio of low pass pyramid [17], morphological pyramid [18], and FSD pyramid [19] have been used for image fusion. However, pyramid transform based fusion methods suffered from blocking effect [20] in the regions where the input images are significantly different. Further, pyramid-transform-based fusion methods do not provide any directional information and have poor signal-to-noise ratio. In contrast to pyramid transforms, wavelet transforms have better representation of detailed features of image; hence, wavelet domain fusion methods provide better results than pyramid-based fusion methods.

The discrete wavelet transform (DWT) is the most commonly used wavelet transform for medical image fusion. A simple DWT-based medical image fusion, which follows weighted fusion rule, has been introduced by Cheng et al. [21]. Another pixel- and region-based multiresolution image fusion for MRI and CT image is discussed in [22]. Several literatures on medical image fusion using DWT can be easily found in [23–30] which use different fusion rules for merging multimodality medical images. Some advanced wavelet families such as contourlet transform, curvelet transform, and nonsubsampled contourlet transform [31] have been used for medical image fusion and it has been stated that these advanced wavelet families have better performance than wavelet transforms. However, these are computationally costly and require huge memory. Further, the study and analysis of DWT for medical image fusion has not been studied well and it still needs attention of researchers.

Since estimation of decomposition levels for a wavelet transform [32] has always been challenging and literatures on multilevel medical image fusion [33] have been a motivation for us to explore DWT for multiscale image fusion, therefore, in this work, we have used DWT for multimodality medical image fusion and presented a new multiscale fusion approach using maximum selection rule. The multiscale fusion approach provides us flexibility to select appropriate fused medical image. The experiments have been performed over several multimodality medical images at multiple scales. The fusion results have been compared with other state-of-the-art fusion methods which include several pyramid- and wavelet-transform-based fusion methods and PCA fusion method. The quantitative analysis of the fusion results has been performed with edge strength (*Q*), mutual information (MI), entropy (*E*), standard deviation (SD), blind structural similarity index metric (BSSIM), spatial frequency (SF), and average gradient (AG) metrics.

The rest of the paper is organized as follows.  Section 2 explains the basics of DWT and its usefulness in image fusion. The proposed fusion method is explained in Section 3. Fusion results and evaluations are given in Section 4. Finally, conclusions of the work are given in Section 5.

## 2. Wavelet Transform and Image Fusion

Recently, wavelet transforms have emerged as a powerful signal and image processing tool which provides an efficient way of fusion using multiresolution analysis [34]. This section explains the basics of DWT and its usefulness in image fusion.

The DWT of a given signal *F*(*x*) is performed by analysis and synthesis of signal using scaling function *ϕ*(*x*) and wavelet function *ψ*(*x*) [35, 36]. The basic equation of multiresolution theory is the scaling equation

(1) $$\begin{array}{c} \phi \left(x\right)=\sqrt{2}\overset{}{\underset{k}{\sum }}l\left(k\right)\phi \left(2x-k\right), \end{array}$$

where *l*(*k*)'s are the approximation or low-pass coefficients and $\sqrt{2}$ maintains the norm of the scaling factor by a factor of two.

The wavelet function *ψ*(*x*) which is responsible for computing high-frequency or detailed coefficients is given by

(2) $$\begin{array}{c} \psi \left(x\right)=\sqrt{2}\overset{}{\underset{k}{\sum }}h\left(k\right)\phi \left(2x-k\right), \end{array}$$

where *h*(*k*)'s are the high frequency or detailed wavelet coefficients.

Signal decomposition is performed using the scaling coefficients *l*(*k*) and the wavelet coefficients *h*(*k*). Forward wavelet analysis of signal *F*(*x*) at any scale *J* is denoted by

(3) $$\begin{array}{c} F\left(x\right)=\overset{}{\underset{k}{\sum }}C\left(j,k\right)\phi_{j,k}\left(x\right)+\overset{}{\underset{j}{\sum }}\sum D\left(j,k\right)\psi_{j,k}\left(x\right), \end{array}$$

where *C*(*j*, *k*) and *D*(*j*, *k*) are scaling and wavelet coefficients at scale *J* and can be computed by following relation:

(4) $$\begin{array}{c} C\left(j,k\right)=\overset{}{\underset{k}{\sum }}l\left(k-2m\right)C\left(j+1,k\right),\\ D\left(j,k\right)=\overset{}{\underset{k}{\sum }}h\left(k-2m\right)C\left(j+1,k\right). \end{array}$$

Reconstruction of signal can be made by combining scaling and wavelet coefficients, and mathematically, it is represented by

(5) $$\begin{array}{c} C\left(j+1,k\right)=\overset{}{\underset{k}{\sum }}C\left(j,k\right)l\left(m-2k\right)+\overset{}{\underset{k}{\sum }}D\left(j,k\right)h\left(m-2k\right). \end{array}$$

The forward and backward analysis of signals provides us the facility to have multiscale signal representations at varying scales. Further, DWT analysis is capable of providing three spatial orientations, namely, horizontal, diagonal, and vertical. This can be denoted by the following combination of scaling and wavelet functions:

(6) $$\begin{array}{c} \phi_{\text{LL}}\left(x,y\right)=\phi \left(x\right)\phi \left(y\right),\\ \psi_{\mathrm{LH}}\left(x,y\right)=\phi \left(x\right)\psi \left(y\right),\\ \psi_{\text{HL}}\left(x,y\right)=\psi \left(x\right)\phi \left(y\right),\\ \psi_{\text{HH}}\left(x,y\right)=\psi \left(x\right)\psi \left(y\right). \end{array}$$

**Figure 1:**
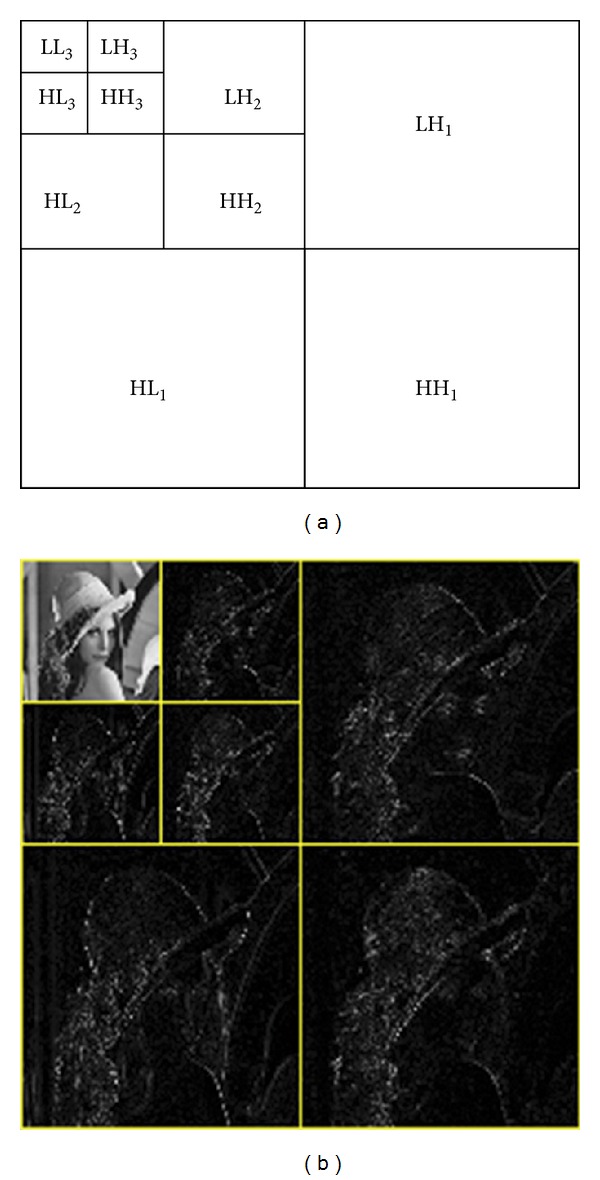
2D decomposition process in discrete wavelet transform (DWT). (a) DWT decomposition up to level 3. (b) Two-level decomposition of Lena image.

The two-dimensional decomposition process using DWT is shown in Figure 1. This can be easily seen that a 2D-DWT provides multiscale representation at different levels.  Figure 1(a) shows the DWT decompositions up to level 3, and Figure 1(b) shows the two-level decomposition of Lena image.

**Figure 2:**
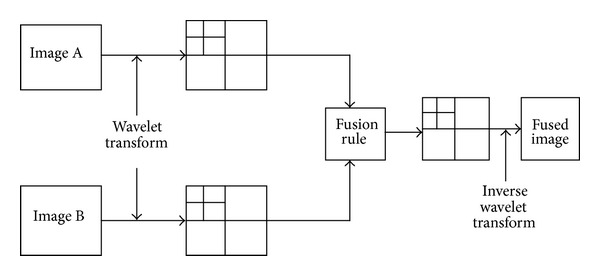
A general image fusion scheme in wavelet domain.

This wavelet decomposition is exploited for image fusion and could be easily understood from Figure 2. The DWT provides an efficient way for performing image fusion at multiple scales with several advantages. These are as follows.

*Locality.* The information of an image is represented by wavelet coefficients, which is local in space and frequency. Thus, for fusion, we can apply fusion rule locally and that would not affect the other portions of the image.

*Multiresolution Analysis. *The image can be represented at different scales, and this allows producing fused images at multiple levels [33].

*Edge Detection.* Wavelet transform can act as local edge detectors. The edges in the image are represented by large wavelet coefficients at the corresponding locations, while noise is generally represented by smaller values. Wavelet transform represents three directional edges: vertical, horizontal, and diagonal. This property helps in preserving the edges and implementation of edge-sensitive fusion methods.

*Decorrelation*. Most of the wavelet coefficients of an image tend to be approximately decorrelated; that is, dependencies between wavelet coefficients are predominantly local. Thus, during the fusion process if there is some change in wavelet coefficients, then generally this would not affect the other portions of image. This allows applying fusion rule on selected wavelet coefficients without affecting other parts of the image.

*Energy Compaction.* In wavelet domain, the most essential information of the image is compressed into relatively few large coefficients, which coincides with the area of major spatial activity (edges, corners, etc.). This property facilitates the implementation of energy-based fusion rules which preserve the salient features of images.

## 3. The Proposed Fusion Approach

The usefulness of DWT made it suitable for medical image fusion, where one wishes to capture all relevant information from a single fused image with reduced cost and storage overhead. The proposed fusion approach follows the framework shown in Figure 2; decomposition was followed by application of fusion rule and reconstruction. We have exploited the concept of multiresolution analysis with multiscale fusion approach. The higher the scale is the more the detailed information is captured from source images to fused image. Since medical images are of poor contrast, more detailed and relevant information should be preserved. Thus, by varying scale, we have flexibility to select appropriate fused image for further operations. For the proposed fusion scheme, we vary the scale from minimum to maximum levels. One of the important issues is the selection of wavelet for decomposition. Regularity, number of vanishing moment, and finite support are the few important criteria for selecting mother wavelet [37]. However, it was shown in [31] that short filter banks for wavelet decomposition are useful and works well for fusion. Also, [38] shows the effectiveness of selecting short length wavelet with a fixed criterion. In both cases, “db3” wavelet has been found suitable for decomposition; therefore, we used “db3” wavelet for the proposed fusion scheme.

The proposed fusion approach is based on the maximum selection scheme as high-valued wavelet coefficients carry salient information such as edges, boundaries, and contours. Therefore, the absolute values of wavelet coefficients have been used for deciding fused wavelet coefficients. For two-source medical images *I*_1_(*x*, *y*) and *I*_2_(*x*, *y*), the steps of the proposed fusion scheme are as follows.

(i) Decompose source images using DWT:

(7) $$\begin{array}{c} W_{1}^{l}\left(x,y\right)=\text{DWT}\left[I_{1}\left(x,y\right)\right],\\ W_{2}^{l}\left(x,y\right)=\text{DWT}\left[I_{2}\left(x,y\right)\right], \end{array}$$

where *W*_1_^*l*^(*x*, *y*) and *W*_2_^*l*^(*x*, *y*) are the wavelet coefficients of source images *I*_1_(*x*, *y*) and *I*_2_(*x*, *y*) at scale *l*.

(ii) Calculate fused wavelet coefficients *W*_*F*_^*l*^(*x*, *y*) at scale *l* by following the expression:

(8) $$\begin{array}{c} W_{F}^{l}\left(x,y\right)=\{\begin{array}{cc} W_{1}^{l}\left(x,y\right), & \text{if}\;\left|W_{1}^{l}\left(x,y\right)\right|\geq \left|W_{2}^{l}\left(x,y\right)\right|\\ W_{2}^{l}\left(x,y\right), & \text{if}\;\left|W_{2}^{l}\left(x,y\right)\right|>\left|W_{1}^{l}\left(x,y\right)\right|. \end{array} \end{array}$$

(iii) Reconstruct fused image *F* ^*l*^(*x*, *y*) at scale *l* using inverse DWT:

(9) $$\begin{array}{c} F^{l}\left(x,y\right)=\text{IDWT}\left[W_{F}^{l}\left(x,y\right)\right]. \end{array}$$

## 4. Fusion Results and Discussions

**Figure 3:**
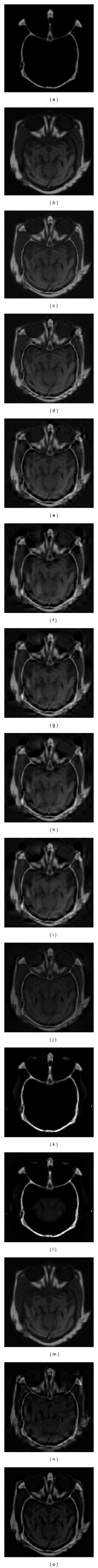
Fusion results for the first set of medical images. (a) CT image, (b) MRI image, (c)–(i) fused images with proposed method from level 2 to level 8, (j) GP method, (k) CP method, (l) RP method, (m) PCA method, (n) DWT with DBSS method, and (o) SIDWT with Haar method.

**Figure 4:**
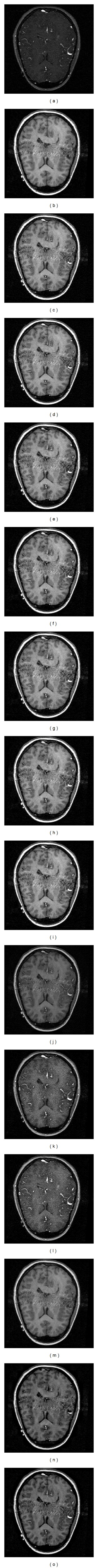
Fusion results for the second set of medical images. (a) MRA image, (b) T1-MR image, (c)–(i) fused images with proposed method from level 2 to level 8, (j) GP method, (k) CP method, (l) RP method, (m) PCA method, (n) DWT with DBSS method, and (o) SIDWT with Haar method.

**Figure 5:**
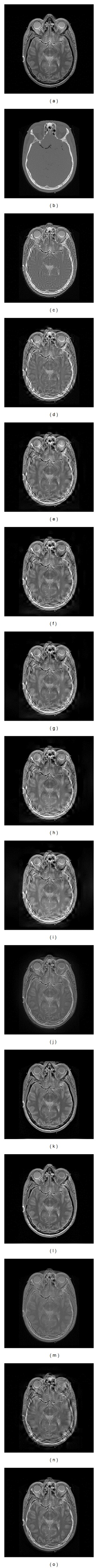
Fusion results for the third set of medical images. (a) MRI image, (b) CT image, (c)–(i) fused images with proposed method from level 2 to level 8, (j) GP method, (k) CP method, (l) RP method, (m) PCA method, (n) DWT with DBSS method, and (o) SIDWT with Haar method.

In this section, we have shown fusion results for the proposed method. The fusion results have been shown for three sets of medical image pairs of size 256 × 256 shown in Figures 3(a), 3(b), 4(a), 4(b), 5(a), and 5(b), respectively. The proposed method has been experimented at multiple scales varying from level 2 to level 8 (maximum level of scale) for these medical images. We have performed subjective and objective comparisons to evaluate fusion results obtained by the proposed method. To perform subjective comparison, we have selected gradient pyramid (GP), contrast pyramid (CP), ratio pyramid (RP), PCA, DWT with DBSS, and SIDWT with Haar fusion methods which are available on http://www.metapix.de/ and provided by Rockinger [39].

For objective evaluation of proposed fusion approach with other state-of-the-art fusion methods, nonreference metrics are required as no ground truth image is available for comparison. Therefore, we have used nonreference metrics, namely, edge strength (*Q*), mutual information (MI), entropy (*E*), standard deviation (SD), blind structural similarity index metric (BSSIM), spatial frequency (SF), and average gradient (AG) for objective evaluation of our work.

The illustration of fusion results is separately given in Sections 4.1 and 4.2 for subjective and objective evaluations, respectively.

### 4.1. Subjective Evaluation

The first set of medical images is brain CT and MRI, shown in Figures 3(a) and 3(b). It can be easily seen that the CT image shows the edgy structure while MRI provides information about soft tissues. The results for proposed multiscale fusion method have been shown in Figures 3(c)–3(i) from level 2 to level 8. These results show the variations in contrast of fused image as level progresses. On comparing the obtained fused images from level 2 to level 8 (shown in Figures 3(c)–3(i)) with GP, CP, RP, and PCA fused images, which are shown in Figures 3(j)–3(m), it can be easily concluded that the proposed method outperforms these fusion methods and has good visual representation of fused image. The fused images with GP, CP, RP, and PCA methods are not able to capture the information from CT and MRI pairs. Further, the proposed method has the better quality than DWT with DBSS and nearly same with SIDWT with Haar fusion methods.

The second set of medical images is magnetic resonance angiogram (MRA) and T1-MR image which is shown in Figures 4(a) and 4(b). The comparison of proposed fusion results with GP, CP, RP, PCA, DWT with DBSS, and SIDWT with Haar fusion methods, shown in Figures 4(c)–4(o), clearly implies that the fused images with proposed method have better quality and contrast in comparison to other fusion methods.

Similarly, on observing the third set of medical images (CT and MRI) and fusion results for these images which are shown in Figures 5(a)–5(o), one can easily verify the fact that again the proposed method has been found superior in terms of visual representation over GP, CP, RP, PCA, DWT with DBSS, and SIDWT with Haar fusion methods.

### 4.2. Objective Evaluation

For objective evaluation of the fusion results, shown from Figures 3–5, we have used seven nonreference fusion metrics: edge strength (*Q*) [40], mutual information (MI) [41], entropy (*E*) [9, 12, 27], standard deviation (SD) [12, 27], blind structural similarity index metric (BSSIM) [33], spatial frequency (SF) [9, 33], and average gradient (AG) [27]. These metrics are well defined in the literature and are used excessively for objective evaluation of fusion results. Higher values of these metrics imply better fused result. We have computed the values of fusion results and tabulated them in Tables 1–3 for fusion results shown in Figures 3–5, respectively.

On observing Table 1, one can easily observe that the fusion measures for proposed multiscale fusion method from level 2 to level 8 have higher values of fusion measures than any of the GP, CP, RP, PCA, DWT with DBSS, and SIDWT with Haar fusion methods. However, the proposed fusion method from level 2 to level 8 has lesser values of SF than CP and RP fusion methods. Also, the proposed method has lesser values of BSSIM than PCA fused image. For these cases, we have performed an overall comparison in Table 1 and it simply states that the proposed multiscale fusion method has better performance for the first set of medical images.

Similarly, observation of Table 2 yields that the proposed fusion method form level 2 to level 8 has higher values of fusion measures than other fusion methods except values of *Q* for PCA and SIDWT with Haar fusion methods and value of BSSIM for PCA fusion method. However, an overall comparison again shows the superiority of the proposed multiscale fusion scheme for the second set of medical images.

Moreover, Table 3 shows the goodness of the proposed fusion method for the third set of medical images except *Q* and BSSIM fusion measures. But again by the same criteria chosen for Tables 1 and 2, the proposed multiscale fusion from level 2 to level 8 has better performance than GP, CP, RP, PCA, DWT with DBSS, and SIDWT with Haar fusion methods.

### 4.3. Combined Evaluation

Since the subjective and objective evaluations separately are not able to examine fusion results, we have combined both, subjective and objective evaluations. The values of fusion measures for fusion results of the first set of medical images (Figure 3) are shown in Table 1. The observations from Table 1 show the variations in the values of SF for CP and RP fusion methods and BSSIM for PCA fusion method. The proposed method from level 2 to level 8 has lesser value of these measures; however, qualitative evaluation of the proposed fusion method from Figure 3 clearly proves the superiority of the proposed method over CP, RP, and PCA fusion methods as these are not able to merge edge and tissue information from source CT and MRI images.

Again, Table 2 shows the higher values of *Q* for PCA and SIDWT with Haar fusion method and value of BSSIM for PCA fusion method, than the proposed fusion scheme for the second set of medical images. However, from Figure 4, it can be easily seen that the proposed method provides better visual representation than any of these fusion methods. Hence, combined evaluation shows the goodness of the proposed multiscale fusion approach.

The fusion measures of Figure 5 are given in Table 3 for the third set of medical images. The measures show the variations in the value of *Q* and BSSIM for the proposed method, and the proposed method has lesser values of *Q* than GP, CP, RP, and SIDWT with Haar fusion methods and lesser values of BSSIM than RP, PCA, and SIDWT with Haar fusion methods. However, qualitative analysis of fusion results shown in Figure 5 clearly shows that GP, CP, RP, and SIDWT with Haar fusion methods have failed to incorporate the features of the source CT and MRI images into one image.

Thus, this combined evaluation for fusion results shown in Figures 3–5 with fusion measures tabulated in Tables 1–3 clearly proves the superiority of the proposed multiscale fusion approach over GP, CP, RP, PCA, DWT with DBSS, and SIDWT with Haar fusion methods.

## 5. Conclusions

In this work, we have proposed a new multiscale image fusion approach for multimodal medical images in wavelet domain and used DWT for proposed fusion method. The multimodal medical images are fused at multiple scales from level 2 (minimum) to level 8 (maximum) scales with maximum fusion rule. The multiscale image fusion method enables the selection of appropriate fused image with better flexibility. To show the effectiveness of the proposed work, we have performed subjective evaluation and objective evaluation of the proposed fusion method with gradient pyramid (GP), contrast pyramid (CP), ratio pyramid (RP), PCA, DWT with DBSS, and SIDWT with Haar fusion methods. The comparative analysis of the fusion results has been performed with edge strength (*Q*), mutual information (MI), entropy (*E*), standard deviation (SD), blind structural similarity index metric (BSSIM), spatial frequency (SF), and average gradient (AG) fusion metrics. Since the subjective and objective evaluations are separately not sufficient for analysis of fusion results, we have performed combined evaluation which proved the superiority of the proposed multiscale fusion approach over GP, CP, RP, PCA, DWT with DBSS, and SIDWT with Haar fusion methods.

## Figures and Tables

**Table 1:** Quantitative evaluation of fusion results for the first set of medical images.

Fusion method	*Q *	MI	*E *	SD	BSSIM	SF	AG
Proposed method—level 2	0.7286	2.1396	5.9335	32.9163	0.5251	9.9138	3.7456
Level 3	0.6675	1.6705	6.0177	32.9062	0.5121	10.3127	3.9258
Level 4	0.6097	1.3475	6.1343	32.9446	0.4931	10.4907	4.0249
Level 5	0.5844	1.3666	6.1673	33.7868	0.4856	10.5698	4.0154
Level 6	0.5871	1.3352	6.2107	33.7941	0.4815	10.5912	4.0377
Level 7	0.5862	1.3137	6.2215	33.7733	0.4805	10.5952	4.0422
Level 8	0.5863	1.3204	6.2225	33.7558	0.4799	10.5947	4.0434
GP	0.5784	1.0243	5.4698	19.7350	0.4793	6.6479	2.3911
CP	0.2542	0.9452	1.9243	33.3962	0.5000	14.5826	2.7348
RP	0.2658	0.9901	3.5655	33.1739	0.4794	14.6512	2.9503
PCA	0.6395	2.6305	5.6220	28.3806	0.5371	6.9945	2.6799
DWT with DBSS	0.4269	1.0342	5.5227	22.8441	0.4429	8.7605	3.1299
SIDWT with Haar	0.7014	1.1510	5.3313	25.6650	0.4816	9.4682	3.3500

**Table 2:** Quantitative evaluation of fusion results for the second set of medical images.

Fusion method	*Q *	MI	*E *	SD	BSSIM	SF	AG
Proposed method—level 2	0.5716	4.1388	6.6049	69.0531	0.6744	27.4395	9.1392
Level 3	0.5607	3.9468	6.5807	69.1204	0.6731	27.5336	9.2222
Level 4	0.5581	3.8518	6.5658	69.1764	0.6718	27.4915	9.1901
Level 5	0.5572	3.8099	6.5218	69.3058	0.6758	27.4752	9.1523
Level 6	0.5568	3.8101	6.5220	69.2432	0.6753	27.4637	9.1471
Level 7	0.5561	3.8052	6.5210	69.2423	0.6753	27.4587	9.1388
Level 8	0.5559	3.8046	6.5222	69.3186	0.6751	27.4539	9.1360
GP	0.5726	3.7534	6.2998	46.0875	0.6938	20.3984	6.2247
CP	0.4355	3.4739	5.8564	47.8375	0.6394	23.2630	7.7518
RP	0.4296	3.5713	5.8947	47.9619	0.6326	23.9939	7.9823
PCA	0.6270	6.5147	6.0242	57.8031	0.7220	20.8370	6.6609
DWT with DBSS	0.4981	2.9846	5.9870	53.5355	0.6302	25.5900	7.8574
SIDWT with Haar	0.6130	3.3325	5.8210	54.3584	0.6614	25.8574	7.8436

**Table 3:** Quantitative evaluation of fusion results for the third set of medical images.

Fusion method	*Q *	MI	*E *	SD	BSSIM	SF	AG
Proposed method—level 2	0.4967	4.0205	5.3671	61.9143	0.7500	23.5420	6.3747
Level 3	0.4873	3.5976	5.4120	61.8504	0.7343	24.0667	6.7283
Level 4	0.4861	3.2489	5.5064	61.5818	0.7091	24.2196	6.1282
Level 5	0.4818	3.0343	5.4945	61.0913	0.7004	24.2190	6.7798
Level 6	0.4768	2.8660	5.6753	61.2172	0.6516	24.0972	6.7355
Level 7	0.4745	2.7483	5.6970	61.7318	0.6372	24.0472	6.6961
Level 8	0.4757	2.7412	6.3419	63.5084	0.5174	24.0370	6.7847
GP	0.5228	3.1159	5.7497	49.6324	0.6742	17.0952	4.4506
CP	0.6475	3.2326	4.5518	55.1066	0.7453	23.8488	6.3681
RP	0.6313	3.6672	4.8964	55.8167	0.7522	24.9349	6.6187
PCA	0.3722	3.8902	4.6139	51.7377	0.7968	12.4883	3.3413
DWT with DBSS	0.4386	3.0704	5.2558	54.1881	0.7076	21.0803	5.8365
SIDWT with Haar	0.6097	3.5023	5.0878	54.2085	0.7667	20.3357	5.4552
